# Nucleolin: a cell portal for viruses, bacteria, and toxins

**DOI:** 10.1007/s00018-022-04300-7

**Published:** 2022-05-03

**Authors:** Fiorella Tonello, Maria Lina Massimino, Caterina Peggion

**Affiliations:** 1 CNR of Italy, Neuroscience Institute, viale G. Colombo 3, 35131 Padua, Italy; 2 Department of Biomedical Sciences, University of Padua, Via Ugo Bassi, 58/B, 35131 Padua, Italy

**Keywords:** Infectious diseases, Snake venom toxins, Cancer, Neurodegenerative diseases, Glycosylation, Phase separation

## Abstract

The main localization of nucleolin is the nucleolus, but this protein is present in multiple subcellular sites, and it is unconventionally secreted. On the cell surface, nucleolin acts as a receptor for various viruses, some bacteria, and some toxins. Aim of this review is to discuss the characteristics that make nucleolin able to act as receptor or co-receptor of so many and different pathogens. The important features that emerge are its multivalence, and its role as a bridge between the cell surface and the nucleus. Multiple domains, short linear motifs and post-translational modifications confer and modulate nucleolin ability to interact with nucleic acids, with proteins, but also with carbohydrates and lipids. This modular multivalence allows nucleolin to participate in different types of biomolecular condensates and to move to various subcellular locations, where it can act as a kind of molecular glue. It moves from the nucleus to the cell surface and can accompany particles in the reverse direction, from the cell surface into the nucleus, which is the destination of several pathogens to manipulate the cell in their favour.

## Introduction

Nucleolin (NCL) is a multi-localized and multifactorial protein involved in countless cellular processes. Its name is due to the fact that it has been discovered, in the seventies, as an abundant component of the nucleolus. Successively however, NCL was observed also in the nucleoplasm and in the cytosol and, since the nineties, it was found also on the cell surface of different eukaryotic cell types. It is a fundamental protein for the life of the cells, and its knockout do not survive, but its level of expression and localization depend on the cycle and state of the cell [1].

It is surprising how many molecular processes NCL is involved in, and with how many types of molecules it interacts. It regulates many facets of DNA and RNA metabolism. It is a histone chaperone and a chromatin remodeler that participate to DNA repair, replication, and recombination [2]. It is involved in the transcription and maturation of ribosomal RNA, and in ribosome assembly and transport [3]. It is also involved in the transcription, splicing, stability, transport, and translation of many mRNA [4]. In the cytosol, it contributes to the microtubules anchoring to centrosomes in interphase cells, and to the microtubule polymerization [5]. On the plasma membrane (PM), it regulates Ras protein assembly and interactions and MAPK signal transduction [6]. On the cell surface, it acts as co-receptor of numerous factors: cytokines, growth factors, and matrix proteins [7]. Because of these and many other functions, NCL is involved in numerous pathologies, its role has been studied especially in cancer and viral diseases [8, 9], but studies on its involvement in neurodegenerative diseases are increasing [10, 11].

**Fig. 1:**
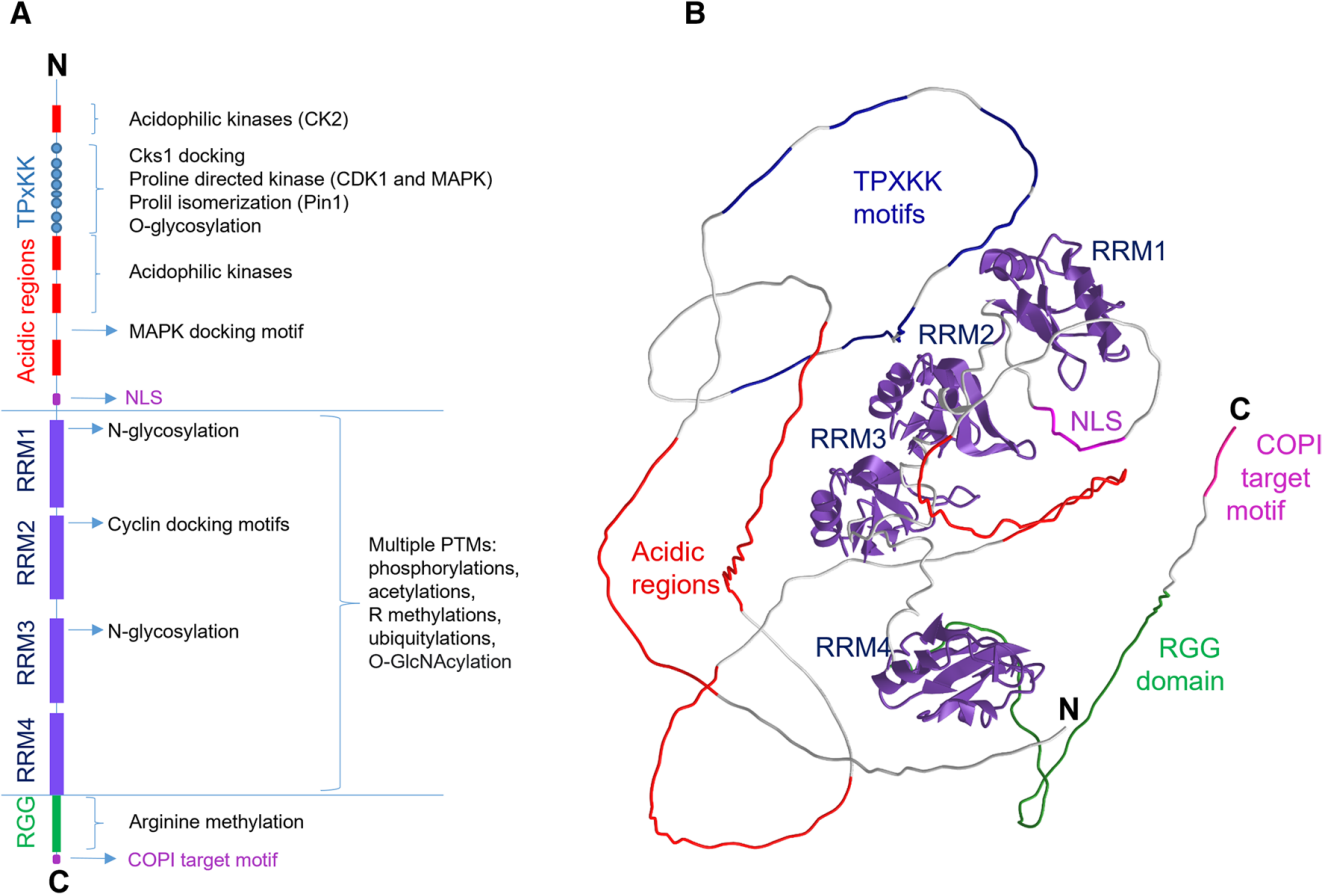
Structure of NCL. **A** Illustration of NCL primary structure. The protein domains have been represented with coloured rectangles, the eight repeated motifs [ST]PxK[KA] (TPxKK in six cases out of eight), have been symbolized with dots. The sites of molecular interactions and modification by PTM enzymes that may be involved in determining the subcellular localisation of NCL have been indicated (see description in paragraphs 2.1 and 2.2, and Table 2). **B** Model of the 3D structure of NCL obtained with AlphaFold [16]. The model confidence of the RRMs domains is high, as the structure the four RMMs domains has been determined by NMR spectroscopy [12, 13]. The representation of the N- and C-terminals has a low level of confidence as these domains are disordered and their structure changes depending on the PTMs. The different domains of NCL are represented in the same colour used in the Fig. 1A

NCL (Fig. 1) is composed of approximately 700 amino acids (exactly, 710 for human NCL and 707 for mouse NCL) and possesses two low complexity, intrinsically disordered domains (IDDs)— one positioned at the N-terminal, and the other at the C-terminal. The N-terminal IDD, 300 amino acids long, is distinguished by the presence of four acidic tracts, the longest of them containing 38 Asp or Glu in a row, without any interruption. These acidic stretches are important in the interaction of NCL with histones that are highly basic proteins. The N-terminal IDD includes also eight repetitions of the motif [ST]PxK[KA] (TPxKK in six cases out of eight), a motif recognized by proline-directed kinases and involved in NCL localization. The central domain is constituted by four RNA-recognition motifs (RRMs), the structure of which was obtained by NMR spectroscopy [12, 13]. RRM is the most common RNA-binding domain (RBD) of higher vertebrates, it is typically composed by about eighty amino acids, and arranged in four-stranded antiparallel beta sheet, among two alpha helices. Each domain recognises between two and eight nucleotides and the combination of multiple domains lengthens the stretch of recognised RNA, which may or may not be continuous [14]. The C-terminal IDD, which consists mainly of an RGG/FGG domain, also contributes to the interaction with RNA [15].

Numerous post-translational modifications have been found in NCL, 179 modification events are reported in the iPTMnet database (October 2021) [17], including phosphorylations, acetylations, ubiquitinations, methylations, and sumoylations. NCL is also a glycosylated protein as shown by Carpentier et al. 2005 [18], and as reported in several works looking for glycosylated antigens present on tumour cells [19–22]. NCL has been reported to be fragmented in cells and, according to Eukaryotic Linear Motif prediction [23], it possesses 12 possible cleavage sites by specific enzymes. In addition, it has 10 potential sites for isomerisation by the prolyl isomerase Pin1 (S/T-P), sites that are regulated by proline-directed kinases. Thanks to its PTMs, NCL can perform its many functions and translocate to different sites in the cell. Moreover, it can interact with different types of molecules: nucleic acids, proteins, carbohydrates, and lipoproteins.

Some twenty review articles have been devoted to NCL since the 2000s (review articles containing the word ‘nucleolin’ in the title indexed in PubMed), most of them on NCL role in cancer. This review addresses the role of NCL as a surface receptor, or rather co-receptor, of pathogens and of pathogenic factors. In the first part of the review, we describe viruses, bacteria, and protein toxins that interact with NCL on the cell surface. In Table 1, we summarise the main data on NCL/pathogens interaction, and we list the inhibitors that have as target this interaction. In the second part we present the main characteristics that make NCL suitable for interaction with so many and different pathogens: first NCL multi-localisation, and the domains, short linear motifs (SLiMs) and PTMs that can regulate it; second NCL glycosylation, which creates additional binding points for viruses and proteins; third NCL phase transition propensity that could give rise to cell surface condensates; and finally NCL involvement in transport pathways from the nucleus to the cell periphery and vice versa. In the concluding session, we discuss the more obscure points of the NCL pathway through the cell, and the possible applications that could be achieved by investigating it further.

**Table 1:** Virus, bacteria, and toxins interacting with NCL on the cell surface

Pathogen or toxin	Other receptors or co-receptors	Mechanism of cell infection or entry	Inhibitors	Refs.
		*Involved NCL Regions/Domains*		
Viruses				
RSV	IGF1R CX3CR1 Annexin II EGFR Lectins TLR4 ICAM-1 HSPGs	Entry mediated by multimeric protein complex formation: (1) IGFR-1-RSV binding; (2) PKCζ activation (3) NCL translocation to PM; (4) NCL-RSV binding; (5) RSV cellular entry	Single-stranded oligonucleotides AS1411 DNA aptamer PKCζ inhibitors Synthetic peptides NCL-specific antibodies NCL–siRNA	[26, 29–31, 75]
		*RBD 1 and 2*		
EVA71	SCARB2, Annexin II, Vimentin, PSGL-1, Fibronectin, Prohibitin, HS proteoglycans, SA-rich proteins	(a) Via endosomes mediated by SCARB2 (lysosome colocalization of EVA71, NCL and SCARB2); (b) Via SCARB2-independent pathway; VCP and UFD1 regulates NCL level facilitating the binding of EVA71 to host cells	NCL–siRNA; NCL-specific antibodies; UFD1 siRNA	[35–37]
HIV	CD4, CXCR4/CCR5, HSPG	Entry mediated by the formation of a multimeric protein complex, in which NCL promotes the translocation of HIV into the cytoplasm	Hb19 and NP63; AS1411; Midkine; Lactoferrin	[39, 42, 43]
		*RGG domains*		
IAV	SA-rich proteins	Via clathrin-mediated endocytosis	NCL–siRNA; NCL-specific antibodies	[45, 46]
HPIV-3	SA-rich proteins; HS	Binding to receptors and fusion with PM	Pretreatment with: NCL-specific antibodies (host cells) NCL protein (virus)	[49]
RHDV	HBGAs	Via clathrin-dependent endocytosis	Competitive blocking peptide NCL-specific antibodies NCL–siRNA	[50]
		*N-terminal residues (aa 285–318) interact with the capsid protein VP60*		
CCHFV	DC-SIGN	Not known	Not tested	[51, 52]
CVB	CAR CD55/DAF	Not known	Not tested	[54]
Bacteria				
Entero-bacteriaceae	Integrin-beta 1	Adhesion to host cells	NCL-specific antibodies	[56, 57]
		*Glucidic modifications*		
*Francisella tularensis*	Mannose receptor; Scavenger receptor A; Complement receptor	Phagocytosis	HB19 NCL–siRNA	[60, 61]
		*RGG domain interacts with F. tularensis EF-Tu*		
Toxins				
*H. pylori Tipα*	Unknown	Stimulated by anti-NCL antibody	NCL–siRNA HB-19 Tunicamycin	[63, 64]
		*Amino acidic region 274–710*		
*B. asper* Mt-II	Unknown	Formation of NCL-Mt-II condensates	Anti-NCL antibody AS1411 NCL–siRNA	[65]
		*The four RRMs and the RGG domain*		
Cathelicidin-BF	Unknown	Interaction with NCL in the cell surface and activation of AMPK-autophagy axis	NCL–siRNA	[68]
LPS	CD14, TLR4 MD2	Interaction with NCL in the cell surface induction of the secretion of inflammatory cytokines	Anti-NCL antibody NCL–siRNA	[70, 71]
Acharan sulfate	Unknown	Interaction with NCL in the cell surface and induction of its translocation to the cytoplasm	Not tested	[73, 74]

The table shows other receptors or co-receptors, beside NCL, involved in the interaction with the pathogen; the mechanisms of cell infection or entry; the regions of NCL involved, if these have been identified; and the inhibitors that act on the NCL–pathogen interaction to prevent the infection or intoxication

## Pathogens interacting with NCL on the cell surface

### Viruses internalized by NCL

Viruses, to carry out their life cycle, must first attach themselves to the host cell, then enter the cell and use the host machinery to create new viruses. Viral binding and entry into eukaryotic cells involve multiple steps and receptors [24]. NCL, expressed on the PM, acts as a co-receptor for the attachment and entry of multiple single-stranded RNA viruses. These include respiratory syncytial virus (RSV), enterovirus A 71 (EVA71), human immunodeficiency virus type 1 (HIV1), various influenza A virus (IAV) subtypes, human parainfluenza virus type 3 (HPIV-3) and rabbit hemorrhagic disease virus (RHDV). NCL is a putative receptor also for Crimean–Congo hemorrhagic fever virus (CCHFV), and B type coxsackieviruses (CVB). Below we report the known receptors of these viruses, we cite the data demonstrating NCL involvement as a viral co-receptor, and if identified, we describe the regions and the PTMs of NCL involved.

**RSV** primarily affects infants and the elderly with a high mortality rate (47%) and can cause lifelong lung problems. To enter into the cells, numerous data demonstrated that RSV interacts with surface receptors, such as annexin II, CX3 chemokine receptor 1 (CX3CR1), epidermal growth factor receptor (EGFR), calcium-dependent lectins, toll-like receptor 4 (TLR4), intercellular adhesion molecule 1 (ICAM-1), glycosaminoglycans (GAG), heparan sulfate proteoglycans (HSPGs), insulin-like growth factor receptor 1 (IGFR-1) and also NCL [25–27]. The attachment and entry into the host cell of RSV occur via two proteins of the virus envelope, called RSV-G and RSV-F; however, RSV-G is not essential as ∆G RSV retains the ability to infect cells [28]. The binding of RSV-F glycoprotein with IGFR-1 triggers the activation of protein kinase C zeta (PKCζ) through which NCL is recruited from the cell nuclei to the PM [29]. RSV infection in vitro is inhibited by anti-NCL antibodies, by NCL–siRNA oligonucleotides [26], by synthetic peptides derived from two 12-mer stretches within NCL–RRM 1 and 2 [30], or by non-coding single-stranded oligonucleotides, able to bind to NCL [31]. Conflicting results were obtained regarding the role of NCL glycosylation in the interaction with the virus, indeed while Holguera et al. [32] has shown that heparin abrogated the binding of RSV to NCL, Tayyari et al. [26] found that heparin does not interfere with the interaction between RSV-F and NCL.

**EVA71** causes hand–foot–mouth disease that mainly affects children. The most severe forms of the infection can lead to encephalitis, aseptic meningitis, pulmonary edema, and acute flaccid paralysis [33]. The main EVA71 receptor is scavenger receptor B2 (SCARB2), a transmembrane protein mainly localised in the PM and in the membranes of lysosome, that is involved in virus internalization and the viral RNA uncoating [34]. Other cell surface factors support EVA71 attachment to the cell surface, such as annexin II, vimentin, P-selectin glycoprotein ligand-1 (PSGL-1), fibronectin, prohibitin, heparan sulfate proteoglycans and NCL. However, they are called as "attachment receptors" because they cannot induce the uncoating of virion particles [35]. Su et al. (2015) identified NCL as one of the main EVA71 interactors, by isolating membrane sialylated glycoproteins interacting with EVA71 particles, from a rhabdomyosarcoma cell line [36]. EVA71 interacts with both sialylated and not-sialylated NCL through the capsid protein VP1. De-sialylation reduced the EVA71 binding to NCL by 30%. NCL and SCARB2 co-precipitate form uninfected cells, and infection with EVA71 increases the amount of NCL associated to SCARB2. An anti-NCL antibody inhibited EVA71 binding to cells, and NCL knockdown by RNA interference decreased EVA71 binding, infection, and production in human cells demonstrating unequivocally that NCL is a co-receptor for EVA71 [36]. Recently, Valosin-containing protein (VCP) and its co-factor UDF1 (ubiquitin recognition factor in ER-associated degradation 1) were found to amplify the binding of EVA71 to host cells by enhancing the expression of NCL [37].

**HIV** is a lentivirus that causes immunodeficiency, leading to death by opportunistic infections or cancer. The attachment of HIV-1 to host cells occurs through virus envelope glycoproteins, the gp120–gp41 complex, and then the virus is internalized through the cellular receptor CD4 and the co-receptor CXCR4/CCR5 for macrophage and T-lymphocyte, respectively [38]. HIV attachment can occur, also in absence of the major receptors, by low-affinity interaction with heparan sulfate proteoglycans and cell surface-expressed NCL [39]. Moreover, NCL forms condensates with CD4, CXCR4, and lipid raft-associated proteins in CD4 + T cells [40, 41]. NCL was isolated in complex also with HIV DNA, viral matrix, and reverse transcriptase, indicating that it follows HIV in all its infection steps [40]. The RGG domains of NCL were identified, by truncated deletion constructs, as the site involved in interaction with HIV [39]. NP63, a polypeptide corresponding to the last 63 amino acids of NCL, and HB-19, a pseudopeptide that binds the RGG NCL domain inhibits viral attachment to the cells [39]. The anti-NCL aptamer AS1411, the heparin-binding growth factors midkine and pleiotrophin, and lactoferrin, all of which interact with NCL and are internalized by NCL on the cell surface, inhibit HIV-1 infection [42, 43].

**IAV** causes respiratory disease with significant morbidity and mortality in humans. This virus binds to sialylated receptors of eukaryotic cells, via their envelope protein hemagglutinin (HA), and are then internalized through different endocytic pathways, but mainly through clathrin vesicles [44, 45]. NCL binds to HA of the influenza virus subtype H1N1 PR8, and it is involved in both attachment and internalization of the virus. NCL depletion causes a decrease in H1N1 PR8 clathrin-mediated endocytosis. RNA interference specific for NCL, and an anti-NCL antibody significantly inhibited virus entry of all influenza A subtypes [46]. NCL may play a role in multiple stages of the influenza virus life cycle. Indeed, NCL interacts also at the nucleolar level with both the viral protein NS1 [47] and with the nuclear protein NP during the viral replication [48].

**HPIV-3** primarily infects lung epithelial cells causing pneumonia and bronchiolitis in infants but also in immunocompromised adults. HPIV-3 binds to sialic acid-containing proteins and to heparan sulfate on the cell surface by its two envelope proteins, hemagglutinin–neuraminidase (HN) and fusion protein (F). HIPV-3 interacts also with NCL expressed on the surface of A549 human lung epithelial cells, and NCL participates in virus internalization. The infection of A549 by HPIV-3 was inhibited by pre-treatment of cells with anti-NCL antibodies, or of the virus particles with purified NCL [49].

**RHDV** is the causing agent of a highly contagious rabbit disease. It binds to histo-blood group antigens (HBGAs) present on the surfaces of cells lining the duodenum and trachea of rabbits. NCL plays a key role in RHDV internalization, as demonstrated by the reduced entry of the virus using a specific NCL antibody or NCL–siRNA oligonucleotides. NCL interacts, through N-terminal residues (aa 285–318), with the capsid protein VP60 causing virus internalization by clathrin-dependent endocytosis. Moreover, a synthetic peptide mimicking the NCL-interacting part of VP60 and containing the DVN motif, reduces virus endocytosis by 60% and partially protects rabbits from the infection [50].

**CCHFV** is transmitted to people through tick bites and causes a disease whose symptoms include high fever, diarrhea, and skin bleeding. CCHFV possesses two envelope glycoproteins, Gn and Gc. A fragment of Gc, residues 180–300, is sufficient to interact with the surface of CCHFV susceptible cells and to co-immuno-precipitate NCL [51]. DC-SIGN, a calcium-dependent [C-type] lectin cell-surface molecule, is another possible entry factor for CCHFV, but no other protein receptor for CCHFV has been identified to date [52].

**CVB** are enteroviruses responsible of different diseases as myocarditis, meningitis, and autoimmune diabetes. All six serotypes of CVB are internalized via the transmembrane protein coxsackievirus and adenovirus receptor (CAR) localized in the tight junctions. The serotypes CVB1, 3 and 5 bind to CD55/DAF (complement decay-accelerating factor) that probably serves as carrier from the apical membrane to the tight junctions [53]. De Verdugo et al. in 1995 found, by virus overlay protein-binding assays and by immunoprecipitation, that NCL interacts with for all six serotypes of the group [54], but other authors argue that NCL does not play an essential role in infection because it binds to pro-virions with greater affinity than to productive CVB viruses [55].

### Bacteria that interact with NCL on the cell surface

NCL interacts with different pathogenic bacteria of the human intestine helping them to adhere to epithelial cells, and with an aerosol spread bacterium, *Francisella tularensis*, promoting the infection of human monocytes.

**Bacteria responsible for enteropathogenic and enterohemorrhagic diarrhoea** attach to intestinal epithelial cells via the protein intimin, a surface bacterial protein, or adhesin, that interact with a bacterial protein, called Translocated intimin receptor (Tir). Tir is injected in the host cell by the type III secretion system, a kind of molecular syringe possessed by this type of bacteria. The interaction between intimin and Tir triggers actin condensation beneath the bacterium, inducing the formation of a pedestal beneath the microorganism. Intimin interact not only with Tir but also with endogenous host proteins including NCL. Different subtypes of intimins exist, belonging to different types of enterobacteriaceae. Intimins alpha, beta, and gamma, belonging respectively to *Escherichia coli* (*E. coli*), *Citrobacter rodentium*, and enterohemorrhagic *E. coli* (EHEC) O157:H7, have all been shown to interact in vitro with NCL and to co-localize with it on the surface of the human epithelial carcinoma Hep-2 cells [56, 57]. Moreover, an antiserum against NCL inhibits the adherence of enterohemorrhagic *E. coli* O157:H7 to Hep-2 cells [56]. NCL and Tir compete for the same region on intimin, but Tir has a higher affinity, suggesting that NCL is involved in the initial adhesion of the bacterium to the epithelial cells. The region of intimin involved in interaction with Tir and NCL is the C-type lectin domain, then probably intimin interacts with glucidic modifications of NCL and Tir. Integrin-beta 1 is another host receptor for intimin-gamma of *E. coli* O157:H7 and, both NCL and integrin beta 1 were observed to be closely associated with the bacteria on bovine and porcine intestinal sections [58].

***Francisella tularensis*** is the cause of tularaemia, an infection also known as rabbit fever that is typically transmitted to humans by rodents. It is a facultative intracellular bacterium, capable of infecting most cell types, but that primarily infects monocyte and macrophages. It induces its phagocytosis by interaction with several host membrane receptors, mainly the mannose receptor for the non-opsonised bacterium, and the class A scavenger receptor, and the complement receptor in the presence of opsonins [59]. After entry into the cells, *F. tularensis* blocks the maturation of the phagosome and exits into the cytoplasm, where it rapidly multiplies. NCL promotes the binding and infection of human monocyte-like THP-1 cells by *F. tularensis* [60] and it co-localizes with the bacterium in the phagosomal compartment [61]. The infection is inhibited by HB-19, a pseudopeptide that binds specifically carboxy-terminal RGG domain of NCL [60], and by RNA interference of NCL [61]. Pull-down assays have demonstrated that NCL interacts with the *F. tularensis* elongation factor Tu (EF-Tu). EF-Tu is one of the most abundant proteins in bacteria and an essential and universally conserved GTPase, necessary for the translation process. Moreover, it is also present on the extracellular surface of the cells where it accomplishes various functions [60, 62].

### Toxins internalized by NCL

NCL is involved in the internalisation of both endogenous and exogenous molecular factors, which can be proteins, either carbohydrates or lipids [7]. Three protein toxins, one secreted by *Helicobacter pylori* (*H. pylori*) and the other two produced by venomous snake glands, interact with cell surface NCL, but also the endotoxin lipopolysaccharide (LPS), and the anti-cancerogenic glycosaminoglycan (GAG) acharan sulfate.

**The *H. pylori* tumor necrosis factor-α (TNF-α)-inducing protein** (Tipα) is secreted by the strains that cause human gastric cancer. Tipα induces the secretion of several inflammatory cytokines and chemokines, including TNF-α, from gastric cell lines and macrophages. Moreover, it induces epithelial–mesenchymal transition and migration of human gastric cancer cells. These and other clues indicate that Tipα is a strong promoter of stomach cancer. Tipα binds to the cell surface and is internalized into the cell cytosol and nucleus. Looking for cell surface receptors of Tipα, the group of Suganuma identified NCL, and that a NCL fragment (274–710) is sufficient to interact in vitro with recombinant Tipα [63]. Moreover, they found that an anti-NCL antibody stimulates Tipα internalization and that pre-treatment with tunicamycin, an inhibitor of N-glycosylation that decreases cell surface NCL, inhibited internalization of Tipα and the induction of TNF-α [63, 64]. Successively the same group demonstrated that the epithelial–mesenchymal transition induced by Tipα is mediated by the binding to cell surface NCL and it is inhibited by anti-NCL–siRNA, or by the pseudopeptide HB-19, that compete for the binding to NCL [7].

***Bothrops asper* myotoxin-II** (Mt-II), a snake venom phospholipase-like toxin that causes muscle necrosis and that shows cytotoxic activity against several type of cells, induces the translocation of NCL on the cell surface and is internalized by NCL in para-nuclear and nuclear region of cells. An anti-NCL antibody and the aptamer AS1411 protect cells from Mt-II cytotoxicity and inhibit the pull-down of NCL by Mt-II. This suggests that the regions of NCL implicated in the interaction with Mt-II are the four RRMs and the RGG domain, the regions recognized by the anti-NCL antibody and that interact with AS1411. Moreover, NCL was found to form a sort of condensates with Mt-II, on the cell surface, when the internalization is inhibited by low temperature [65]. The mechanism of cell death induced by *B. asper* myotoxin-II has not been clarified, but two homologue Lys49 toxins, bothropstoxin-I of *Bothrops jararacussu* and BnSP-6 of *Bothrops pauloensis*, induce the death of different cancer cell lines by autophagy and apoptosis [66, 67].

**Cathelicidin-BF** is an antimicrobial peptide isolated from snake *Bungarus fasciatus* venom that strongly inhibits melanoma cells growth. LZ1, a peptide derived from cathelicidin-BF, less toxic than the full cathelicidin, is a potent growth inhibitor of pancreatic cancer cells. LZ1 binds to and colocalize with cell surface NCL and it induces its internalization, resulting in an autophagy-dependent cell death via the activation of adenosine monophosphate-activated protein kinase (AMPK) [68].

**LPS**, a large molecule composed of lipid and a polysaccharide, is the major component of the outer membrane of Gram-negative bacteria. LPS binding to receptors present on many cell types, but mainly monocytes/macrophages, dendritic cells, and leukocytes, induces the secretion of inflammatory cytokines. Recognition of LPS on the cell surface takes place via a complex formed by the proteins CD14, toll-like receptor 4 (TLR4) and the small soluble protein called MD2, but it involves also many other proteins depending on the cells and on the exact composition of the LPS molecule [69]. Cell surface NCL is involved in the activation of THP-1 human monocytes by LSP and the pre-treatment of these cells with anti-NCL antibody significantly inhibited the secretion of inflammatory cytokines [70, 71]. Moreover, LPS colocalizes with NCL on the surface and cytoplasm of primary alveolar rat macrophages, and NCL knockdown by siRNA inhibits LPS internalization and the macrophages activation [72].

**Acharan sulfate** is a GAG isolated from the body of the giant African snail Achatina fulica. GAGs are linear polysaccharides made up of repeating units of negatively charged disaccharides and, depending on their composition, they can have different pharmacological activities. Acharan sulfate showed antiangiogenic, anticoagulant and antimitogenic activity, and little cytotoxicity on various cancer cells. NCL was identified as the cell surface molecule interacting with acharan sulfate, in mouse Lewis lung carcinoma cells and in A549 human lung adenocarcinomas, by affinity chromatography and colocalization experiments. Moreover, acharan sulfate was found to induce the translocation of NCL from nucleus to cytoplasm of A549 cells [73, 74].

### NCL is a multi-localized and an unconventional secreted protein

NCL is found primarily in the nucleolus of cells where it plays a role in many steps of ribosome production, from the transcription of ribosomal DNA to the ribosome assembly. It also moves into the nucleoplasm where it participates in several functions, including DNA repair, DNA transcription and RNA splicing. Although in smaller amounts, NCL is also found in the cytosol where it is part of ribonucleoprotein complexes, and it stabilizes and regulates the subcellular localization and translation of several mRNAs [1, 76, 77]. The subcellular mRNA localization given by NCL determines the growth and size both of neurons and cycling cells [78] and the local synthesis of mTor, whose mRNA is transported by NCL, and controls axonal local translation in nerve injury [79]. In some type of cells, mainly proliferating and/or cancerogenic cells, NCL is present on the cell surface, too. Alternatively, NCL can translocate to the cell surface upon stimulation of membrane receptor [1, 76, 77]. In the following paragraphs, we describe the characteristics of NCL that influence its intracellular localization, and the route that NCL can take to exit the cells.

#### NCL interactions, SLiMs and PTMs that affect its intracellular localization

**Fig. 2:**
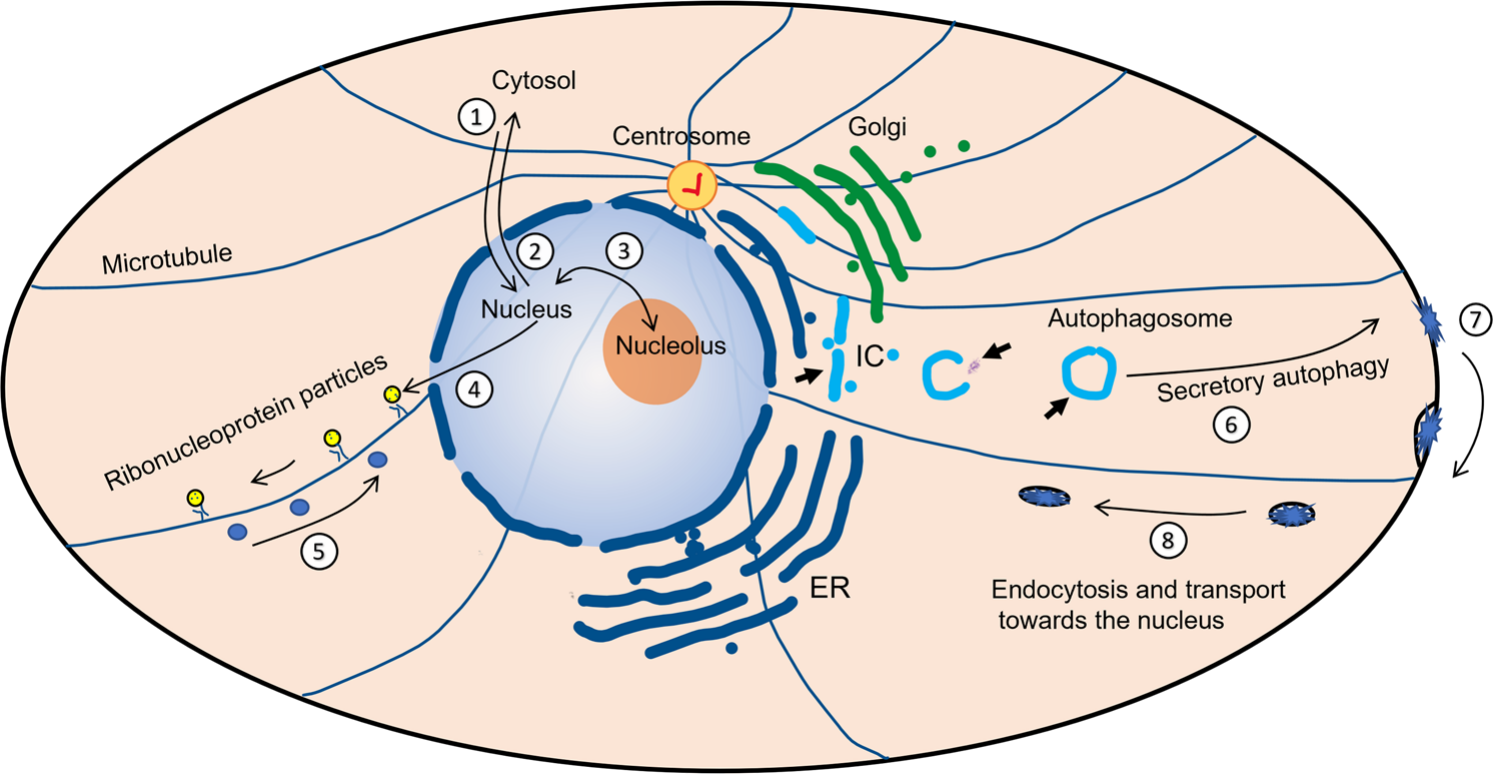
Intracellular movement of NCL. After the synthesis, NCL is transported to the nucleoplasm, through the nuclear pores, thanks to a nuclear localization sequence (**1**). Several PTMs, S/T phosphorylation by CDK1, R methylation by PRMT5, K acetylation by p300, induce the re-localization of NCL in the cytosol (**2**). The nucleolar localization (**3**) is determined by the interaction of NCL with other nucleolar proteins and with nucleic acids. In the nucleus and nucleolus, NCL participates to the formation of ribonucleoproteins that are transported, by kinesin motion, along microtubules towards the cell borders (**4**). NCL has been shown to bind beta-importin mRNA and, after beta-importin translation, to be part of a retrograde movement of protein complexes towards the centre of the cell (**5**). NCL can move to the cell surface through an unconventional secretion process (**6**) that has not been clarified but that can involve translocation of the protein in the intermediate compartment (IC or ERGIC), autophagy or chaperon-mediated autophagy (bold arrows) followed by docking and fusion of the autophagosome to the cell membrane. In the cell surface, NCL, in the presence of different ligands, can induce the clusterization of receptors and trigger an endocytic process (**7**). From the periphery of the cell, vesicles and/or molecular particles can move towards the nucleus escorted by NCL; however, the path that takes these particles to the nucleus is still unknown, it can involve retrograde transport through the Golgi and ER, or maybe through the intermediate compartment (**8**). It is still a mystery how some transmembrane receptors, interacting with NCL and with viruses, can reach the nucleoplasm, whether through the nuclear pores or by insertion into the nuclear membrane and subsequent release, in micro-vesicles or in some other forms of lipid particles

The subcellular localization of a protein depends on the interactions it establishes with other molecules in the cell, interactions that may vary depending on the cellular state. Many protein–protein interactions occur between globular domains and SLiMs which fit into their binding pocket. SLiMs are typically composed of 3–4 conserved amino acids, are present in IDDs or in exposed loops, and their binding affinity for globular domains is shaped by PTMs. In this paragraph, but also in the following ones, in Figs. 1 and 2 and in Table 2, we describe the SLiMs and PTMs of NCL that are known to have, or could have, an impact on its localization.

**Table 2:** Domains, SLiMs, and PTMs involved in NCL subcellular localization

Site	PTMs (involved enzyme)	Effect	References
N-terminal acidic regions (24–43, 145–171, 184–210)	S/T phosphorylation by acidophilic kinase (CK2)	Movement of NCL during the cell cycle; shuttling between the cytoplasm and the nucleoplasm	[85, 137]
TPxKK repeat region (58–135), Cks1 docking sites (74–124), docking site for cyclins in RMM2 (393–405), docking site for MAPKs (217–227)	S/T phosphorylation by proline-directed kinases (CDK1 and MAPKs), prolyl isomerization (Pin1)	Movement of NCL during the cell cycle; shuttling between the cytoplasm and the nucleoplasm	[85, 138]
K88	Acetylation	Co-localization with splicing factors	[139]
Nuclear localization sequence (NLS) amino acids279-298	–	Basic residues that are recognized by importin-alpha, an adaptor protein necessary for the interaction with importin-beta that guides proteins through the nuclear pores	[139]
T84, T92, T105, T106, T113	Possible O-glycosylation with N-acetylgalactosamine	Glycosylation of secreted NCL	[18]
T301, T438, T587, S619, T641	Possible glycosylation with O-linked β-N-acetyl glucosamine (O-GlcNAcylation)	It occurs in the cytosol and nucleus and regulates protein localization based on the cellular metabolic state	[89]
N317, N492	N-glycosylation	Necessary for the unconventional secretion of NCL	[18]
RMM domains	K acetylation (p300), S/T phosphorylation, arginine methylation (PRMT5), ubiquitylation	Interaction with nucleic acids; nucleolar localization	[84, 88]
C-terminal RGG region	Arginine methylation (PRMT5)	Interaction with nucleic acids; involved in nucleolar localization; translocation from nucleus to the cytoplasm; interaction with membranes	[83, 88]
Unknown	Phosphorylation by PKCζ	PTM necessary for the unconventional secretion of NCL stimulated by RSV	[29]
Penta-peptide motifs recognized by Hsc70 (motif start: 374, 376, 420, 544, 554, 638, 705)	The motifs can be activated by phosphorylation and/or acetylation	Translocation to auto-phagosomes mediated by Hsc70. Possible step in the unconventional secretion of NCL	[92, 95]
Di-Lysine signal responsible for COPI-mediated retrieval from post-ER compartments (706–710)	–	Could be involved in the unconventional secretory and in the endocytosis pathways of NCL	[140]

*CK2* casein kinase 2, *Cks1* cyclin-dependent kinase subunit 1, *CDK1* cyclin-dependent kinase 1, *MAPKs* mitogen-activated protein kinase, *Pin1* Peptidyl-prolyl cis–trans isomerase NIMA-interacting 1, *PRMT5* Protein arginine N-methyltransferase 5, *PKCζ* protein kinase C zeta, *COPI* coat protein complex

NCL transport to the nucleus is due to a bipartite nuclear localization sequence (NLS) present at the end of the N-terminal disordered region (amino acids 279–298) [80]. This region contains basic residues that are recognized by importin-alpha, an adaptor protein necessary for the interaction with importin-beta that guides proteins through the nuclear pores. Within the nucleus, acetylation at K88 determines NLC co-localization with splicing factors, while the main localization of NCL, that in the nucleolus, is regulated by the interaction, of its RRMs and GAR regions, with RNA and ribosomal proteins [81, 82]. The methylation of arginine residues in the C-terminal domain and the numerous and various PTMs of the RMM domains affect NCL interaction with nucleic acids and consequently NCL localization [83, 84].

NCL does not have a nuclear export sequence and its return to the cytoplasm is regulated, during the cell cycle, by the phosphorylation of multiples sites by casein kinase 2 (CK2) in the acid region, and by cyclin directed kinase 1 (CDK1 also known as cdc2) at the basic TPxKK repeats. In particular, the phosphorylation by CDK1 retains NCL in the cytosol [85]. The phosphorylation process by CKs and CDKs is very complex and intersects with the activities of other PTM enzymes in a hierarchical mode [86, 87]. In the case of CDKs, the activity is regulated by interactions with cyclins and their regulatory subunits, and with the activity of prolyl isomerase 1 (Pin1). The phosphorylation process of NCL has unfortunately not yet been studied, but interestingly docking sites for cyclins are present in the RRM2 of NCL, indicating that this region can be necessary for the phosphorylation process of the TPxKK repeats.

Recently, CITED2, (CBP/p300-interacting transactivator with E/D-rich carboxy-terminal domain-2), a transcription co-factor that regulates fundamental cell processes, was found to be necessary for the methylation and acetylation of NCL in the nucleus, by recruiting PRMT5 and p300. Following these modifications, NCL is translocated to the cytoplasm [88]. Moreover, a high-throughput screening analysis evidenced five threonine residues (T301, T438, T587, S619, T641), in human NCL, that are modified by O-linked β-N-acetyl glucosamine, a PTM that occurs in the cytosol and nucleus and regulates protein localization based on the cellular metabolic state [89].

#### NCL secretion pathway

NCL does not possess a signal peptide for the secretion, and specific inhibitors of the canonical pathway of secretion, brefeldin A and monensin, do not inhibit its externalization, so the mechanism that brings it to the cell surface is part of the unconventional mechanisms of secretion [90]. The unconventional mechanisms of secretion can be divided into two main groups: those involving the direct crossing of the PM with the help of other proteins or of some type of lipids, and those involving the loading of the protein in vesicles that subsequently fuse with PM [91]. Experiments conducted on NCL secretion have shown that: first, it requires the intervention of the chaperonin Hsc70 [92]; second that NCL is contained in vesicles [90]; and third that secretion requires NCL N-glycosylation, a PTM catalysed by enzymes that are known to reside in the ER and Golgi [93]. This latter finding, apparently in contradiction with the fact that NCL follows an unconventional secretion, can be explained by assuming that NCL is translocated into compartments containing glycosyl-transferase enzymes and from which NCL can reach the surface without passing through the classical secretion pathway.

The type of NCL-containing vesicles has not been identified, but other proteins that undergo unconventional secretion are loaded in autophagosomes by micro-autophagy [94], or by chaperon-mediated autophagy (CMA) with the help of Hsc70, which recognises specific motifs in cytosolic proteins [95]. Autophagosomes, as an alternative to fusing with lysosomes to degrade their contents, can reach the PM and release proteins or vesicles outside the cell, in a process called secretory autophagy [96]. Hsc70 is a protein required for both microautophagy and CMA, as well as for NCL localisation at the cell surface, so NCL could be translocated to autophagosomes via one of these two pathways. Moreover, NCL possesses seven motifs (KFERQ-like motifs) than can be recognized by Hsc70 (Table 2) and trigger CMA [95]. In alternative, some unconventional secreted proteins, as interleukin-beta 1, galectin-3 and others, have been observed to be translocated, by the chaperon Hsp90 and the transmembrane protein TMED10, in the ER–Golgi intermediate compartment (ERGIC or IC) [97]. Since in melanoma cells, galectin-3 forms complexes with glycosylated NCLs within cells and on the cell surface [98], it is conceivable that they take the same route to get out of cells. From the ERGIC, proteins can move to the Golgi, or to the ER, or follow a third, yet not well-defined, route to the PM without passing through the Golgi, possibly involving autophagosomes, as ERGIC has been proven to contribute to phagosome formation [99].

The C-terminal domain of NCL could be involved in the secretion steps. Indeed, the RGG region of NCL is involved in interaction with the membranes and it is necessary to the localization in the PM [100]. After the RGG domain, there is an interaction motif with COPI, a protein complex that coats vesicles involved in the retro-transport from Golgi to the ERGIC and ER, and that is involved also in the autophagosome formation [101].

Based on this information, it is possible to speculate that the most likely route NCL can take to exit the cell is the translocation to the ER/ERGIC, where it can be glycosylated, followed by a pathway along Golgi cisternae, and then back to the ERGIC via COPI vesicles. After that, an autophagy secretory pathway, or other pathway that transport proteins from the ERGIC to the PM, could release NCL outside of the cell (see Fig. 2). Indeed, according to the most recent studies, the intermediate compartment no longer appears to be just a junction between the ER and the Golgi, but a true organelle that maintains its structure during the cell cycle and performs independent signalling and cell traffic functions [102].

### Glycosylation of cell surface NCL increase its ligand-binding capacity

Cell surface NCL is an extensively glycosylated protein [18, 98]. Moreover, many glucidic antigens (glycoepitopes) characteristic of cancer cells were found covalently attached to NCL: Tn antigen [19, 98], Sialyl-Lewis A [22, 98], 9-O-acetylated sialic acid [21], and fucose [103]. Carpentier et al. have identified in NCL two N-glycosylation sites (N-GlcNac), in domains RRM1 (N317) and RRM3 (N492), and two O-glycosyl sialylation sytes (O-GalNac substituted with sialic acid) in unknown positions [18]. The predicted O-GalNac sites are five according to Carpentier et al. while the neural network NetOGlyc—4.0 identifies 24 potential sites [104], including all serines and threonines that precede a proline. NCL additionally possesses potential modification sites with glucosamine glycan [105].

Glycosylation can explain NCL ability to interact with numerous pathogens. Both viruses and bacteria, and even several toxins, interact with glycans on the cell surface (Varki 2017). Sialic acid, linked to cell surface glycoproteins and ganglioside, acts as receptor from many viruses, and could be the responsible of the spill over from mammals to man [106]. The glucidic component of NCL has been involved in the interaction with influenza virus and EVA71 that utilize sialic acid in their infection process, and with RSV that interacts with GAGs [32, 46]. The region of intimins, the enterobacteriaceae adhesins, involved in interaction with NCL and with Tir (paragraph 1.2) is the C-type lectin domain, suggesting that glycosylation of Tir and NCL can be important in this interaction [57]. Snake toxins that interact with cell surface NCL, described in the paragraph 1.3, and many endogenous proteins that are internalized via NCL [7] are positively charged and have the characteristic of binding to negatively charged carbohydrates such as heparin.

In addition to being glycosylated, NCL also acts as a lectin, indeed it can bind to carbohydrates, which is why it is involved in the interaction and internalization of LPS and the GAG acharan sulfate (paragraph 1.2). This property of NCL can be involved in its relationship with viruses as, in the process of viruses binding and entry, the interactions between glycosylated virus proteins and lectins are important too [107].

### Multivalency of NCL and the formation of biomolecular condensates on the cell surface

Biomolecular condensates (BMCs), or membrane-less organelles (MLOs), are dynamic concentrates of molecules that are not enclosed by a lipid bilayer. The formation of many MLOs is possible, thanks to spontaneous and reversible liquid–liquid phase separation (LLPS), or phase transition processes, in which multiple weak attractive interactions between molecules allow their segregation from the surrounding environment, to promote specific activities in isolated spaces [108, 109]. BMCs can contain thousands of different proteins, some proteins are characteristic of some condensate, while others are widespread and can transfer from one condensate to another. Moreover, some proteins are fundamental to the condensate, and their deletion leads to the disintegration of the MLO, while others are accessories and can be eliminated without consequences on the MLO existence. Proteins that are fundamental to the condensate have the characteristic of being multivalent, i.e., to be able to establish many molecular interactions, and are often composed of both modular domains, with a precise structure and function, and of IDRs, plastic traits enriched in adhesive SLiMs [110]. NCL, as described in the first paragraph, is a multivalent protein composed of four modular RBDs and of two IDRs, and it is a protein essential for the biggest MLO of the cell, the nucleolus, in fact its inactivation leads to nucleolar disruption [111]. NCL participates also to other intracellular molecular clusters that are considered BMCs [112], e.g., the nuclear speckles where are concentrated the pre-mRNA splicing factors [113], the RNP transport granules [114], the nucleation of microtubules [115].

LLPS mediates the formation not only of three-dimensional MLOs such as nucleoli, but also of two-dimensional condensates on the PM, where multivalent proteins, both intra- and extracellular, are required for transmembrane signalling, activation of endocytosis, or formation of focal adhesions [116]. Multivalent proteins, on the inside of the PM, connect transmembrane proteins with cytosolic adaptor to activate signal cascades or anchorage to the cytoskeleton, while, on the outside of the cell, they link together external ligands with transmembrane receptors. The cluster of the proteins LAT, Grb2, and Sos1 is an example of an inner membrane condensates, it leads to the activation of Ras proteins, GTPases that trigger the MAPK signalling pathway [117]. Cytosolic NCL could be involved in a similar condensate as it binds to ErbB transmembrane receptors [118] and Ras proteins [6] and regulates MAPK signalling transduction.

Galectin-3 (Gal-3) is one example of extracellular multivalent protein that promotes LLPS on the cell surface. It is an unconventional secreted monomeric lectin characterized by the presence of one carbohydrate recognition domain (CDR), flanked to an intrinsically disordered proline-rich N-terminal domain. On the cell surface, Gal-3 induces receptor clustering and a clathrin-independent endocytosis. When Gal-3 binds to cell surface glycoproteins it oligomerizes, thanks to interaction between the N-terminal and the CDR domain, forming dynamic LLPS, or LLPS-like, condensates that can be reversed by lactose [119, 120]. NCL, being an interactor of Gal-3, could be a component of condensates promoted by this protein [98]. Moreover, since NCL, as Gal-3, is a multivalent protein capable of interacting with itself and with different kinds of molecules, we can infer that the complexes formed by NCL on the cell surface, such as those formed with IGF1R [29], or with *B. asper* Mt-II [65], could be LLPS phenomena too.

### NCL provides a physical link between the nucleus and the cell periphery and vice versa

NCL is part of those ribonucleoprotein complexes that leave the nucleus and follow the microtubules to localise in different areas of the cell where they give rise to translation of mRNA. Moreover, NCL has been shown to bind beta-importin mRNA and, after beta-importin translation, to be part of a retrograde movement of protein complexes towards the centre of the cell. These movements of molecular complexes from the centre to the periphery of the cell and vice versa, constitute a mechanism for monitoring and control the cell dimension [78]. NCL also moves to the outer surface of the cell, probably after translocation to vesicular organelles, followed by fusion of intracellular vesicles with PM (see paragraph 2.1.2). From the cell surface, after forming complexes with external ligands and membrane receptors, NCL returns to the interior of the cell via endocytosis, and in some cases, NCL was found to accompany internalised external factors all the way into the nucleus [7, 121].

The nucleus contains the cellular genome in the form of chromatin, a complex of nucleic acids and proteins with a complex three-dimensional organization that determines the gene expression, i.e., which parts of DNA are transcribed, and which are not. Moreover, the nucleus contains ribonucleoprotein complexes that regulate the modification of RNA after transcription, such as ribosome formation and splicing. The organization of chromatin and of the various ribonucleoprotein complexes is regulated by PTMs, and by the numberless proteins localized in the nucleus, many of which have different subcellular localizations and are transported to the nucleus only under special circumstances. Some of the proteins that are transported to the nucleus are known for their nuclear function, e.g., transcription factors that move from the cytoplasm to the nucleus. Other proteins instead moonlight in the nucleus performing functions other than those that are considered the main ones [122, 123].

Several pathogens target the cell nucleus for manipulating the cell to facilitate their own replication [24]. Retroviruses have evolved different mechanisms to get their RNA, or the transcribed DNA, into the nucleus to integrate with the cell genome [124]. Influenza viruses replicate their RNA into the nucleus [125]. Moreover, viruses that replicate their nucleic acids into the cytoplasm also need to reach the cell nucleus with some viral factors, for altering the gene expression in their favour [126]. Many bacteria likewise produce factor that reaches the nucleus to manipulate the cells, e.g. TipA of *H. pylori* (paragraph 1.3), and that have been defined ‘nucleomodulins’ [127]. Given this need to reach the nucleus by numerous types of pathogens and factors released by them, it is likely that NCL has been selected as a cell surface interactor, direct or indirect, because it is part of a trafficking pathway leading from the cell surface to the nucleus.

How the transport from the cell surface to the nucleus occurs is still unclear, several pathways could be involved. In the case of viruses, after endocytosis and maturation of the endosomes, the viral capsid or its ribonucleoproteins penetrate the cytosol, and successively they move towards the nucleus along microtubules. Viral ribonucleoproteins and small capsids can reach the nucleoplasm through the nuclear pore complexes [24]. The HIV-1 capsid, that has a size larger than the NPC central channel, was thought to release its contents into the cytosol, but recently, it was found to reach intact the nucleus through the NPC and release the viral DNA near its genomic integration site [128, 129]. Or in alternative, HIV particles gathered around MTOC after travelling along the microtubules, were shown to enter the nucleus via nuclear envelope rupture and restoration [130]. NCL, besides its involvement in the membrane docking and endocytosis of several viruses, could have a role also in the trafficking to the nucleus, as happens in the case of other ribonucleoprotein particles.

In the case of EGFR and IGF1R, membrane receptors that interact with cell surface NCL, it has been shown that the transport to the nucleus requires COPI proteins and Sec61 [131]. COPI proteins are known to form coatomers around vesicles for retrograde transport from Golgi cisternae to ER, but in a few cases, they resulted necessary for endocytosis processes too [132–134]. Moreover, COPI proteins have recently been shown to be involved in transport along neuronal microtubules, in RNP trafficking and in the local translation of mRNA, processes in which NCL plays a key role [135, 136]. This suggests that the C-terminal SLiM motif of NCL, which is required for interaction with COPI proteins, could be involved in the formation of vesicles, or other types of molecular complexes, moving along microtubules from the nucleus to the cell periphery and vice versa (Fig. 2).

## Conclusion

The ability of NCL to act as a receptor or co-receptor for numerous types of pathogens is explained by its multivalence, i.e. its ability to interact with different types of molecules, proteins, nucleic acids, carbohydrates, and lipids. Multivalence, modulated by the PTMs, gives to NCL the ability to move between various subcellular locations, from the centre to the periphery of the cell. NCL is unconventionally secreted and on the cell surface, it can take part to molecular condensates involving both membrane receptors and extracellular factors. Cell surface NCL is both N- and O-glycosylated and these modifications increase both the ability of NCL to form multimers, and its ability to interact with viruses, bacteria, and toxins. In addition, NCL is a component of a pathway leading from the cell surface to the nucleus, and this is an excellent reason for it to be selected as a co-receptor, as the nucleus is the target of many pathogens to modulate the cell in their favour.

Many steps are still unclear on the movements and interactions established by NCL: the PTM modifications of NCL and their hierarchical order; the NCL secretion pathway and consequently the glycosylation it undergoes, where it happens and how it is controlled; the properties of the condensates that form NCL on the cell surface; the pathway taken by NCL to carry molecules from the cell surface to the nucleus. Trafficking from the cell surface to the nucleus is still a mystery involving not only NCL but also several transmembrane proteins with which it interacts. It is unknown if the proteins enter the nucleus via vesicles, or alternatively as molecular complexes containing phospholipids, back-transported through the nuclear envelope or the nuclear pores.

Understanding the mechanisms of NCL re-localization and of the interaction with different extracellular factors is important not only to complete the characterization of this multifactorial protein, but also to develop drugs able to prevent the entry of pathogens, in particular viruses. On the other hand, the study of internalization and the effect of pathogens, and of cytotoxins, is useful for the development of anticancer therapies since this NCL is enriched on the surface of cancer cells. Such cell surface localisation and the ability to bind and internalise different pathogens, strongly suggest that NCL may also interact with the tumour-associated microbiome, the study of which emerged a few years ago, and whose role in cancer initiation, progression or regression is under discussion [141, 142]. Not least, NCL, given its role in trafficking from the cell surface to the nucleus, may serve as a portal for the transport to the nucleus of gene therapy drugs.

## Data Availability

Not applicable.
